# Intrauterine Adenosine Administration for the Treatment of Fetal Supraventricular Tachycardia in a Fetus With Aortic Stenosis

**DOI:** 10.7759/cureus.42931

**Published:** 2023-08-04

**Authors:** Alexandria Weymon, Katherine T Huebner, Julie Sommerfield, Marcos Cordoba, Vivian Romero

**Affiliations:** 1 Maternal-Fetal Medicine, Michigan State University College of Human Medicine, Grand Rapids, USA; 2 Pediatric Cardiology, Helen DeVos Children's Hospital, Grand Rapids, USA; 3 Maternal-Fetal Medicine, Corewell Health Medical Group/Michigan State University, Grand Rapids, USA

**Keywords:** adenosine, intrauterine, congenital aortic stenosis, supraventricular tachycardia, fetal tachyarrhythmia

## Abstract

Fetal tachyarrhythmia and aortic stenosis (AS) both disrupt fetal hemodynamics, leading to congestive heart failure, hydrops, and intrauterine demise. Traditional transplacental treatments for fetal supraventricular tachycardia (SVT) include digoxin, flecainide, and sotalol. However, the treatment of fetal SVT in the setting of AS has not been described, particularly in cases of refractory SVT. We present a case of a 27-year-old nulliparous female carrying a fetus with fetal AS diagnosed at 25 weeks of gestational age (GA). The patient was not a candidate for in utero valvuloplasty. During ultrasound monitoring at 32 and 6/7 weeks of gestation, fetal SVT with a heart rate of 230-260 beats per minute (bpm) was diagnosed. Maternal digoxin was initiated, and sotalol was subsequently added. Due to persistent fetal SVT and a worsening cardiac function, the patient was treated with direct adenosine administration via cordocentesis successfully terminating the fetal arrhythmia. Despite continued transplacental treatment with digoxin and sotalol throughout the course of pregnancy, the fetal SVT recurred at 35 and 5/7 weeks of gestation prompting delivery. Our case illustrates the use of direct intrauterine adenosine as a novel treatment for refractory fetal SVT in the setting of congenital aortic stenosis and concern about progression to fetal hydrops and fetal demise.

## Introduction

Sustained fetal tachyarrhythmia (heart rate of above 200 beats per minute {bpm}) impedes ventricular diastolic filling and thereby reduces stroke volume and cardiac output. In the setting of normal cardiac anatomy, prolonged tachycardia and the consequent reduction of cardiac output can cause hydrops and fetal demise. While fetal tachyarrhythmias are rare, the most commonly diagnosed is supraventricular tachycardia (SVT) [1]. The conventional treatment of fetal SVT consists of the maternal administration of digoxin, flecainide, and/or sotalol [2]. Congenital aortic stenosis (AS), caused by a hypoplastic and/or dysplastic aortic valve, increases left ventricular (LV) systolic pressure, resulting in hypertrophy and scarification within the left ventricle. The thickened ventricle develops poor diastolic function, reduced stroke volume, and decreased cardiac output that can progress to hypoplastic left heart syndrome (HLHS) [3-5]. Intrauterine valvuloplasty for fetal AS has been performed in selected patients and centers [6]. Fetal SVT refractory to digoxin and sotalol is uncommon, and management, particularly in cases of congenital heart defects, is not well described.

We present a case of fetal AS in which in utero valvuloplasty was not indicated but was later complicated by SVT refractory to appropriate transplacental antiarrhythmic therapy with digoxin and sotalol. Given the concern for potential fetal congestive heart failure and hydrops, shared decision-making among the mother and maternal-fetal medicine, pediatric cardiology, and neonatal teams prompted the treatment of fetal SVT with direct adenosine via cordocentesis and the continuation of maternal digoxin and sotalol to prevent relapse. This combined regimen offers a novel therapeutic approach to fetal SVT in the setting of structural heart defect. This case was previously presented as an oral presentation at the 2022 American Institute of Ultrasound in Medicine (AIUM) Annual Meeting on March 13, 2022.

## Case presentation

**Figure 1:**
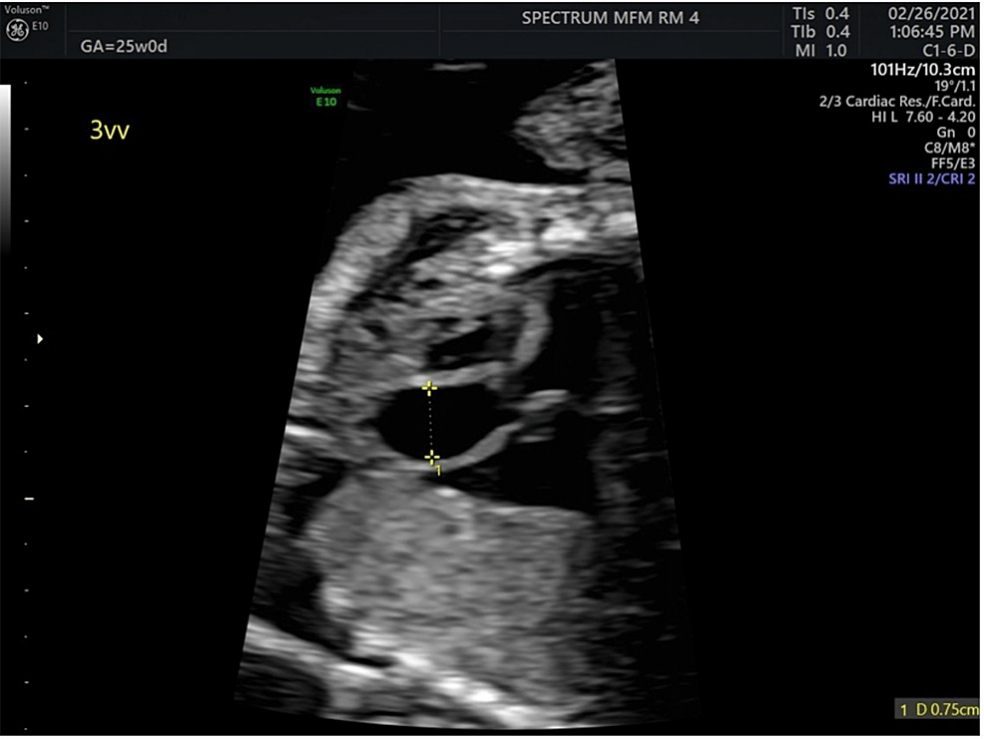
Initial echocardiogram at 25 weeks of gestational age Left ventricular outflow tract (LVOT) view demonstrating a dilated ascending aorta diameter measuring 7.99 mm, immediately distal to the LVOT at the initial echocardiogram at 25 weeks of gestation

**Figure 2:**
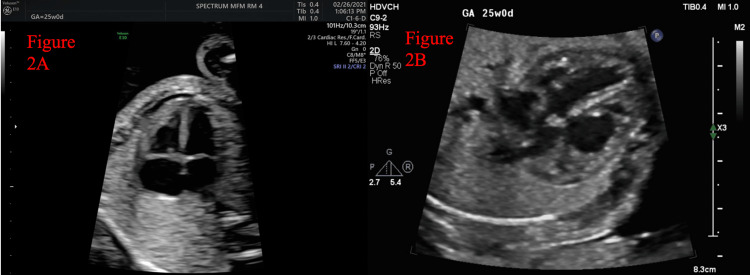
Apical four-chamber and transverse view at 25 weeks of gestational age Apical four-chamber view (A) and transverse view (B) demonstrating an absence of ventricular hypertrophy, endocardial fibroelastosis (EFE), or pericardial effusion at the initial echocardiogram at 25 weeks of gestation

**Figure 3:**
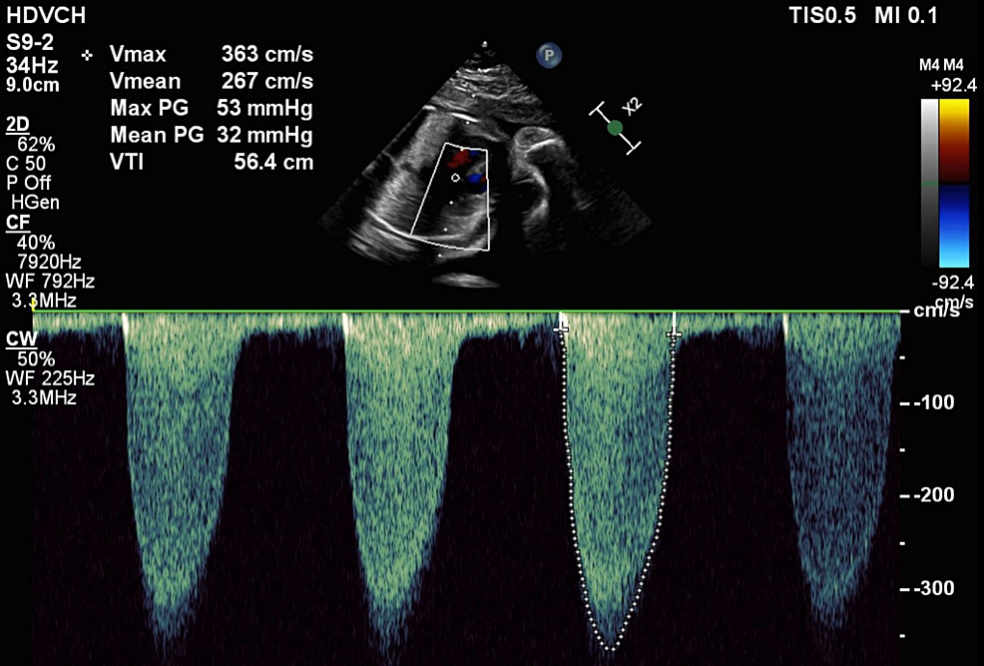
Doppler echocardiogram at 25 weeks of gestational age Pulsed wave Doppler demonstrating markedly increased pressures across the aortic valve with a maximum peak gradient of 53 mmHg and a mean of 32 mmHg (peak velocity: 3.6 m/s)

**Figure 4:**
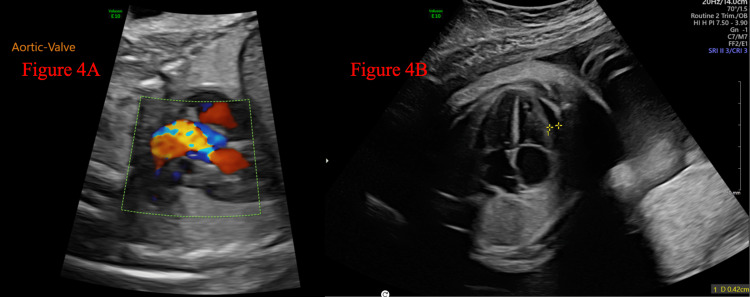
Echocardiogram at 32 and 6/7 weeks of gestation (A) LVOT view demonstrating turbulent blood flow through the narrowed aortic outflow tract. (B) Apical four-chamber view of the fetal heart minimal pericardial effusion measuring 4.2 mm on echocardiography performed at 32 and 6/7 weeks of gestation LVOT: left ventricular outflow tract

**Figure 5:**
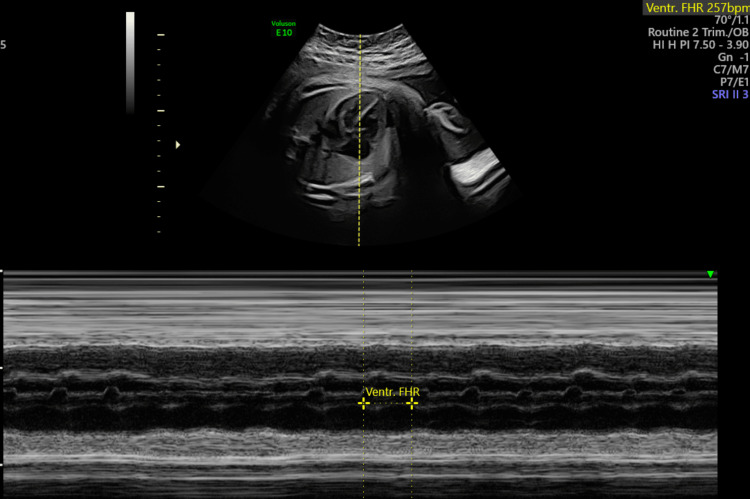
M-mode image at 32 and 6/7 weeks of gestation M-mode recording in SVT with 1:1 AV relation; fetal HR of 257 bpm seen at 32 and 6/7 weeks of gestation SVT, supraventricular tachycardia; AV, atrioventricular; HR, heart rate; bpm, beats per minute

A 27-year-old nulliparous female was referred to our Fetal Care Center due to an increased fetal aortic diameter noted on routine ultrasound at 25 weeks of gestation. A fetal echocardiogram showed dysplastic and thickened aortic valve leaflets with restricted motion and a dilated ascending aortic diameter measuring 7.99 mm, confirming AS (Figure 1). LV systolic function was preserved with only mild LV hypertrophy and no endocardial fibroelastosis (EFE) (Figure 2). The initial peak gradient was 53 mmHg, with a mean gradient of 32 mmHg (peak velocity: 3.6 m/s) (Figure 3). The patient was not a candidate for intrauterine fetal aortic valvuloplasty because of preserved LV function and the absence of EFE. The fetus was monitored with serial echocardiography at one-to-two-week intervals to assess LV systolic function. An echocardiogram at 28 weeks of gestational age (GA) showed new moderate mitral valve regurgitation, bidirectional flow across the foramen ovale, and increased LV hypertrophy. An echocardiogram performed at 32 and 6/7 weeks of gestation detected EFE for the first time, a minimal pericardial effusion (Figure 4), and fetal SVT between 230 and 250 bpm prompting hospital admission (Figure 5). The mother was given a loading dose of 0.5 mg digoxin, followed by 0.25 mg every six hours (a total of 1.5 mg for 24 hours). Fetal SVT resolved overnight but recurred the following day. Sotalol 160 mg twice daily was added but failed to convert the arrhythmia. Repeat fetal echocardiogram demonstrated poor systolic function and an increasing pericardial effusion. Considering the rapid development of the pericardial effusion and the worsening cardiac function, there was a serious concern for intrauterine demise. The in utero administration of adenosine via cordocentesis was offered as an alternative treatment to promptly cardiovert fetal SVT while avoiding maternal toxicity. After carefully evaluating the risks and benefits, the patient consented to the fetal procedure.

The dosage of adenosine was calculated based on 0.2 mg/kg and an ultrasound estimated fetal weight of 2.38 kg. Under continuous ultrasound guidance, an IV bolus of adenosine 0.48 mg was administered directly into the umbilical vein with a 1 mL saline flush. SVT persisted without pause. A second dose of adenosine 0.48 mg was administered with a 1 mL saline flush, causing a brief pause in the fetal arrhythmia followed by a resumption of sinus rhythm (115 bpm). Fetal ventricular systolic function was depressed for a few beats before rapidly improving. No acute fetal or maternal adverse events were observed other than maternal subjective palpations overnight. The mother was monitored on telemetry for the remainder of her admission and showed no arrhythmia or corrected QT (QTc) prolongation. The adult cardiology service evaluated the mother and found no signs of cardiac compromise. Fetal echocardiography on day 3 showed significant improvement of the pericardial effusion and no evidence of hydrops. The mother was discharged after five days and continued digoxin 0.125 mg once daily, sotalol 160 mg twice daily, and weekly fetal echocardiography until delivery.

Fetal SVT recurred briefly at 35 and 3/7 weeks of GA, although the echocardiogram did not show effusions or evidence of hydrops. Given the GA, the induction of labor was recommended. The mother delivered a female infant vaginally at 35 and 5/7 weeks of GA without complications. Apgar scores were 8 and 9 at one and five minutes. The neonatal team was present at delivery. The infant underwent cardiac surgery on day 1 of life and is doing well at two years after two cardiac procedures and multiple ablations.

## Discussion

Fetal AS and SVT are independently associated with life-threatening hemodynamic abnormalities that can cause intrauterine fetal demise. Fetal echocardiography has markedly improved the ability to diagnose and treat AS, as well as to screen for hydrops fetalis and hypoplastic left heart syndrome, producing a window of opportunity for early intervention and the prevention of intrauterine demise [2,6]. Conventional treatment options to prevent negative sequela of fetal AS include premature delivery and postnatal surgery, transplacental digoxin, or intrauterine fetal aortic valvuloplasty [4,6]. However, not all fetuses with AS will develop HLHS, and the complication rate for intrauterine surgery is high. Mäkikallio et al. [4] suggest that fetuses with AS at the highest risk of developing HLHS exhibit retrograde flow through the transverse aortic arch and have early evidence of moderate-to-severe LV dysfunction. Both features were absent in this fetus and, thus, not a candidate for intrauterine aortic valvuloplasty.

SVT in the setting of AS is particularly uncommon. Fetal SVT that is refractory to standard treatment with transplacental digoxin and sotalol is infrequent but can lead to rapidly progressive hydrops due to decreased cardiac output. Considering the multiple cardiac disorders in this case, the risks of heart failure and intrauterine death were high. A recent multicenter trial that sought to standardize fetal SVT treatment found that a regimen of maternal digoxin and sotalol was effective and tolerable in a vast majority of patients and was followed by infrequent relapses [2]. However, a small percentage of fetuses may develop recurrent and potentially lethal tachyarrhythmia within two weeks despite appropriate treatment. Literature that describes the direct fetal administration of adenosine to interrupt SVT is sparse. One case report described direct adenosine treatment in a 23-week fetus with incessant reentrant SVT and hydrops fetalis [7]. A similar dosage of adenosine was administered via the umbilical vein and achieved a normal sinus rhythm within seconds. The rationale was that the majority of fetal tachyarrhythmias are secondary to reentrant pathways and that adenosine is safe and efficacious in pediatric patients [7], making it an ideal drug of choice in a fetus with a refractory arrhythmia. Cordocentesis was chosen as the route of administration because the extremely short half-life of adenosine precludes transplacental drug passage [7,8]. However, the author noted that the fetus had several brief episodes of tachycardia within minutes after administration, validating the use of adenosine as therapy versus prophylaxis. Thus, after sinus rhythm is quickly achieved, maintenance therapy with digoxin can prevent recurrence.

In our case, the fetus had preserved LV systolic function but later developed mitral valve regurgitation and EFE in the setting of AS, increasing the risk of hydrops fetalis. This risk dramatically increased after the onset of SVT. Despite the prompt initiation of standard antiarrhythmic therapy, SVT persisted, fostering the decision for more aggressive interventions. The well-established risks of cordocentesis include umbilical hematomas, infection, cardiac depression, and premature rupture of membranes. Administering adenosine directly to the fetus via cordocentesis bypasses maternal metabolism, allowing a therapeutic effect on the fetus with a rapid onset of action. Therefore, the risks of direct fetal adenosine must be carefully weighed against the benefits and only be considered in refractory cases where the risk of fetal demise is high.

The recommended dosage of adenosine in pediatric patients is 0.2 mg/kg [8], with an estimated fetal weight determined by ultrasonography. Two 0.48 mg doses of fetal adenosine spaced by 1-2 minutes were required to restore a normal sinus rhythm. There was no change in the tachyarrhythmia from the first dose. After the second dose, cardiac contractility was depressed briefly, returning to normal sinus rhythm within one minute. The post-procedural monitoring of the mother and fetus disclosed no objective adverse reactions or new cardiac anomalies. Digoxin and sotalol were continued prophylactically throughout the course of the pregnancy. This combined regimen prevented further episodes of SVT and consequent fetal hydrops and cardiac decompensation despite comorbid AS. In addition, the regimen allowed the mother to safely deliver her baby vaginally in the late preterm period and obviated significant complications associated with prematurity.

## Conclusions

Our case demonstrates the direct administration of intrauterine adenosine to cardiovert fetal SVT refractory to the mainstays of therapeutic intervention in a fetus with congenital structural heart disease and at high risk for worsening cardiac function. The absence of maternal or fetal adverse events after direct fetal adenosine treatment is encouraging. However, a limitation is that this approach to SVT management has not been investigated on a larger scale or in patients with different demographics or comorbidities. Furthermore, this is a single retrospective case report without a control group. In the future, larger studies including a risk-benefit analysis of treating fetuses with adenosine via cordocentesis would be most beneficial to more broadly establish safety and efficacy.
